# Assessing the performance of international pre‐exposure prophylaxis (PrEP) eligibility guidelines in a cohort of Chinese MSM, Beijing, China 2009 to 2016

**DOI:** 10.1002/jia2.25653

**Published:** 2020-12-20

**Authors:** Eric W Hall, Liming Wang, Xiaojie Huang, Patrick S Sullivan, Aaron J Siegler

**Affiliations:** ^1^ Department of Epidemiology Rollins School of Public Health Emory University Atlanta GA USA; ^2^ Emory University/Blued Beijing China; ^3^ Center for Infectious Diseases Beijing You’an Hospital Capital Medical University Beijing P.R. China; ^4^ Department of Behavioral, Social and Health Education Sciences Rollins School of Public Health Emory University Atlanta GA USA

**Keywords:** HIV prevention, PrEP, men who have sex with men, sexually transmitted infections/diseases, Asia<‐Region, public health

## Abstract

**Introduction:**

PrEP is a powerful HIV prevention tool, and locally relevant eligibility criteria are necessary to optimize the prevention impact of PrEP. We assessed performance of existing national and international PrEP eligibility criteria to predict future HIV seroconversion among MSM in Beijing, China.

**Methods:**

Participants were MSM aged ≥18 years who enrolled in a cohort study between July 2009 and March 2016. Participants completed HIV testing, syphilis testing, and a questionnaire on recent sexual health behaviours at each follow‐up visit and were followed until HIV seroconversion or dropout. We assessed PrEP eligibility at the most recent follow‐up visit prior to the final study visit. Participants were classified as indicated for PrEP (or not) based on criteria from guidelines from Europe, Korea, South Africa, Taiwan, the United Kingdom, United States and the World Health Organization. To compare guideline performance, we calculated sensitivity, specificity, Youden’s Index (YI), Matthew’s Correlation Coefficient (MCC), F1 scores and diagnostic odds ratios. For each guideline, performance measures were compared to random allocation of PrEP by randomly selecting a proportion of participants equal to the proportion indicated.

**Results:**

There were 287 (17∙3%) incident HIV seroconversions among 1663 MSM. The number of men indicated for PrEP from different guidelines ranged from 556 (33∙4%) to 1569 (94∙2%). Compared to random allocation, sensitivity of algorithms to predict seroconversion ranged from slightly worse (−4∙7%) to 30∙2% better than random. However, in absolute terms, none of the sensitivity values increased by more than 11% when compared to random allocation. For all guidelines, specificity was not meaningfully better than random allocation. No guidelines had high binary classification performance measures.

**Conclusions:**

The performance of international indication guidelines in this sample was only slightly better than random allocation. Using such guidelines to screen out MSM self‐identifying as interested in PrEP could lead to misallocation of resources and to good candidates for PrEP being denied access. For settings in which international guidelines perform poorly, alternative indication approaches should be considered.

## 1 Introduction

When taken correctly, human immunodeficiency virus (HIV) pre‐exposure prophylaxis (PrEP) has been shown to be effective in preventing the risk of HIV seroconversion by over 90% in men who have sex with men (MSM) [1]. However, because costs of the medication and clinical care are high, PrEP strategies are most cost‐effective when they target high‐risk individuals [2, 3, 4, 5]. Several sets of clinical guidelines and tools have been developed to assess risk of acquiring HIV among MSM in different countries or regions [6, 7, 8, 9], but their predictive ability to identify persons at high‐risk of HIV infection can differ between populations [10]. To achieve optimal public health impact, PrEP initiation guidelines should differ between target populations and be informed by HIV risk assessment measures that perform best in the specific population of interest.

Since it was first approved by the United States (US) Food and Drug Administration in 2012, PrEP has now been approved for HIV prevention in 20% (38/193) of the UN member nations and that number is expected to increase [11]. A notable exception is China, in which tenofovir disoproxil fumarate (TDF) / emtricitabine (FTC) has been approved for treatment of HIV infection but not received regulatory approval for use as prevention. In China, 95% of new HIV infections now occur through sexual contact and annual HIV incidence among MSM is estimated to be higher than 3% [12]. A recent modelling study indicated that over the next two decades, 170,000 to 320,000 new HIV infections would be prevented if PrEP were introduced and coverage reached 50% among MSM in China [13].

When a national PrEP programme is implemented, countries must determine who should be indicated to receive the intervention. Countries can draw from implementation guidelines that have been developed by other countries and international health organizations, but to the extent informative data are available, should be specific to local standards of care and epidemic characteristics. HIV risk depends both on individual behaviours and on the prevalence of unsuppressed HIV infection in partner pools [14], and local data collection or STI screening practices might yield different information for decisions about PrEP indications. For example, the US Public Health Service (USPHS) MSM PrEP initiation guidelines rely on results from routine laboratory testing for syphilis and nucleic acid amplification tests three‐site testing for chlamydia and gonorrhoea. However, in China it is not common practice to test regularly for chlamydia and gonorrhoea. Guidelines from both USPHS and WHO include criteria that require unprotected sex with multiple partners in the past six months for PrEP to be indicated. In comparison, guidelines from South Africa and Korea have less restrictive entry requirements and only require MSM to be sexually active. If applied to a population of MSM not experienced with PrEP, such as countries that have not yet established PrEP indication guidelines, these different initiation criteria would be expected to change both the number and the characteristic of persons indicated for PrEP, potentially having a population‐level impact on PrEP scale‐up.

In this analysis, we assessed the performance of a number of different international PrEP eligibility criteria in predicting future HIV seroconversion in a cohort of Chinese MSM in Beijing. We then compare the performance of each set of guidelines in terms of sensitivity and specificity, to random allocation of PrEP irrespective of behaviour.

## 2 Methods

### 2.1 Participants

Data for this analysis are from a subset of previously published prospective cohort study that aimed to estimate HIV incidence among MSM in Beijing [15]. For that study, all participants were predominantly recruited through flyers at venues that are frequented by MSM. We used an electronic informed consent procedure, conducted within the study survey platform. To be eligible for enrolment, respondents had to be aged ≥18 years, report anal sex with a male partner in the past six months, and be HIV negative at their baseline cohort visit. TDF/FTC for PrEP was not readily available during the study period because it was not approved for use as HIV prevention in China at the time of the study, so PrEP use was not assessed in the study questionnaire. All participants were enrolled at You’An hospital between 2007 and March 2013. At the baseline enrolment visit and all subsequent study visits, participants were tested for HIV and syphilis, and completed a questionnaire on sexual health behaviours that collected information on partners and condom use frequency during the previous two months. Participants were encouraged to return for a study visit every three months and were followed until HIV seroconversion or study discontinuation. Ethical approval was provided by China National Centers for AIDS/STD Control and Prevention (NCAIDS) (KX180117492), which is registered with the US Office for Human Research Protections, IRB0000227, and has a Federal wide Assurance (FWA00002958).

### 2.2 Study instruments and analytic cohort

To be considered for this analysis, participants were required to have at least two study visits. First, we identified the final study visit (seroconversion or final HIV‐negative test). Next, we limited the cohort to only include participants that had at least one additional visit that occurred between six and twenty‐four months prior to their final study visit. At the most recent study visit within this look‐back interval, participants were assessed for PrEP eligibility according to eight different sets of PrEP guidelines: European AIDS Clinical Society (EACS) [16], Korean Society for AIDS (KSA) [17], Southern African HIV Clinicians Society (SA) [18], Taiwan Centers for Disease Control [19], British HIV Association (UK) [20], United States Public Health Service clinical guidelines (USPHSC) [21] and risk score (USPHSR) [6, 22], and the World Health Organization (WHO) [23]. Behavioural data and syphilis test results were operationalized to match each specific criteria from each PrEP eligibility assessment tool as defined in Table 1. Several sets of guidelines include criteria that we were unable to assess due to unavailable data, such as the use of drugs or alcohol during sex or history of post‐exposure prophylaxis (PEP) use. If a participant reported an HIV‐positive partner, we assumed for guidelines translation purposes that the partner was not virally suppressed. For persons in the dataset with missing data on condom use, we assumed condom use was less than 100%. For each set of guidelines, all participants were categorized as indicated for PrEP when they met the respective criteria and not indicated for PrEP when they did not.

**Table 1:** Operationalization of international PrEP eligibility guidelines with You’An cohort data, Beijing, China

Guidelines	Classification in You'An data
European AIDS Clinical Society (EACS)	
No HIV infection AND	Inclusion criteria
Have sex with men AND	Number of male sex partners >0
Inconsistent condom use with use with casual partners OR	Number of non‐fixed partners >0 and condom use <100%
Inconsistent condom use with use with HIV+ partners not on treatment OR	Condom use with fixed HIV‐positive partner <100%
Recent STI	Reported diagnosis of any STI or positive syphilis TPPA test
Use of PEP	*Not in data*
Korean Society for AIDS (KSA)	
No HIV infection AND	Inclusion criteria
Sexually active	Number of sex partners >0
Southern African HIV Clinicians Soceity (SA)	
No HIV infection AND	Inclusion criteria
Sexually active AND	Number of sex partners >0
HIV‐positive partner that is not confirmed virologically suppressed OR	Number of HIV‐positive partners >0
Partner of unknown status OR	*Not in data*
Recent STI OR	Reported diagnosis of any STI or positive syphilis TPPA test
Multiple sex partners OR	Number of sex partners >1
History of inconsistent condom use OR	Condom use for vaginal or anal sex <100%
Commercial sex work OR	Number of sex work partners >0
Recent PEP use OR	*Not in data*
Sex while under the influence of alcohol or drugs	*Not in data*
Taiwan Centers for Disease Control	
No HIV infection AND	Inclusion criteria
At least one episode of condomless anal intercourse OR	Number of anal sex partners >0 & condom use <100%
Recent STI (last 6 months) OR	Reported diagnosis of any STI or positive syphilis TPPA test
Condomless anal sex with HIV‐positive partners OR	Number of unprotected anal sex acts with HIV‐positive partners >0
At least one episode of Chemsex OR	*Not in data*
Use of PEP	*Not in data*
British HIV Association (UK)	
No HIV infection AND	Inclusion criteria
Condomless anal sex in last 6 months (and ongoing) OR	Number of anal sex partners >0 & condom use <100%
HIV‐positive partner, unless partner is on treatment.	Number of HIV‐positive partners >0
United States Public Health Service ‐ clinical (USPHSC)	
No HIV infection AND	Inclusion criteria
Any male sex partners in last 6 months AND	Number of male sex partners >0
Not in a monogamous partnership with a recently tested, HIV‐negative male partnerAND	Number of sex partners that are not monogamous >0
Anal sex without condoms in last 6 months OR	Number of anal sex partners >0 & condom use <100%
STI in last 6 months OR	Reported diagnosis of any STI or positive syphilis TPPA test
Ongoing sexual relationship with HIV‐positive male partner	Number of fixed HIV‐positive male partners >0
United States Public Health Service – risk score (USPHSR)	
No HIV infection AND	Inclusion criteria
Determined to be high risk by risk assessment score	Calculated risk score >9
World Health Organization (WHO)	
No HIV infection AND	Inclusion criteria
Vaginal or anal sexual intercourse without a condom with more than one partner OR	Number of vaginal and anal partners >1 and condom use <100%
STI in last 6 months (self‐report or lab) OR	Reported diagnosis of any STI or positive syphilis TPPA test
PEP use in last 6 months OR	*Not in data*
Sexual partner has HIV and is not on ART or virally suppressed	Number of HIV‐positive partners >0

### 2.3 Statistical analysis

**Figure 1:**
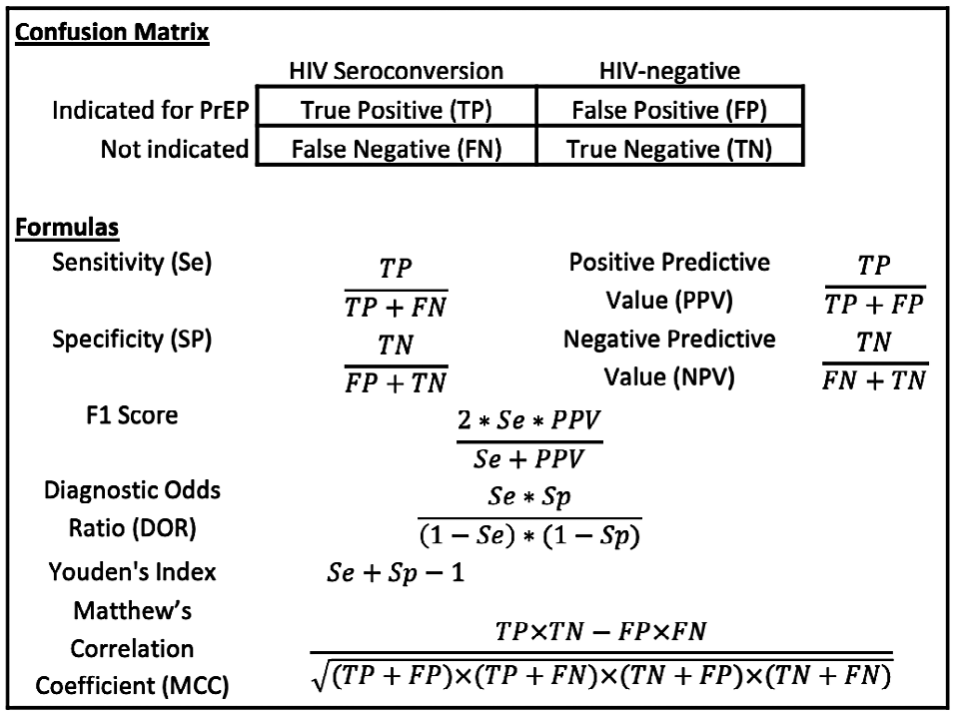
Formulas for classification assessment measures.

Demographic data and follow‐up time were summarized by HIV seroconversion status. To compare the performance of PrEP guidelines, we calculated the sensitivity, specificity, positive predictive value, negative predictive value and corresponding exact binomial confidence intervals for each set of guidelines (Figure 1 provides formulas for each metric). In addition, we calculated several statistics that are often used in machine learning to assess the performance of binary classifiers [24]. F1 score summarizes sensitivity and positive predictive value and ranges from 0 to 1 [25]. The diagnostic odds ratio (DOR) ranges from 0 to infinity and is the odds of a participant being indicated for PrEP if they are a seroconversion versus the odds of a participant being indicated for PrEP if they are not a seroconversion. Youden’s Index (YI) ranges from 0 to 1 and evaluates the guidelines ability to avoid misclassification, where a value of zero indicates the guidelines are unable to discriminate between groups and a value of one indicates perfect classification [26]. Matthew’s correlation coefficient (MCC) is the correlation between predicted and observed classification and ranges from −1 (complete disagreement) to 1 (perfect prediction) [27].

We sought to understand the relative utility of each set of guidelines compared to an alternative where no selection criteria were used, but a proportion of individuals identical to the proportion for that guidelines was designated for PrEP, but assigned randomly rather than according to the guideline score. The classification performance of each set of guidelines was thus compared to the random allocation of PrEP as a counterfactual eligibility criterion. For each set of guidelines, we randomly selected a subset of participants equal to the total number of participants that were indicated for PrEP using the guidelines. The randomly selected participants were classified as PrEP eligible and we calculated the same classification assessment measures described earlier. This random draw was repeated 10,000 times to calculate a point estimate and 95% bootstrap credible interval for each classification assessment measure. To assess the performance of each set of guidelines against random allocation, we calculated the percent change between the point estimate from each classification measure and the point estimate of random allocation.

## 3 Results

There were 1,663 participants eligible for this analysis and 287 (17∙3%) new HIV seroconversions were documented (Table 2). The median number of days between PrEP eligibility assessment and final study visit was 251 (interquartile range, IQR: 216 to 323) among participants who seroconverted and 276 (IQR: 223 to 385) among participants that remained HIV‐negative. Overall, the median age at PrEP assessment was 30 years (IQR: 26 to 37) and the large majority of participants (95∙4%, n = 1587) were living in Beijing province. About half (n = 934, 56∙2%) of participants reported being single during their PrEP assessment study visit and the internet was the most commonly reported venue for meeting sex partners (n = 806, 48∙5%). There were no meaningful demographic differences between participants who seroconverted and participants that remained HIV negative.

**Table 2:** Demographic characteristics, by HIV seroconversion status, of a cohort of MSM enrolled from 2009 to 2016, You’An Hospital, Beijing, China

	Overall (n = 1663)		HIV+ (n = 287)		HIV− (n = 1376)	
Oldest date	7/20/2009		7/29/2009		7/20/2009	
Most recent date	3/23/2016		3/4/2016		3/23/2016	
Days between visits (median; IQR)	271.0	221 to 373	251.0	216 to 323	276.0	223 to 385
Age in years at PrEP assessment (median; IQR)	30.0	26.0 to 37.0	29.0	25.0 to 36.0	30.0	26.0 to 37.0
	**n**	**%**	**n**	**%**	**n**	**%**
Current living province						
Beijing	1587	95.4	273	95.1	1314	95.5
Hebei	54	3.2	11	3.8	43	3.1
Tianjin	9	0.5	1	0.4	8	0.6
Other	13	0.8	2	0.7	11	0.8
Marriage status						
Single	934	56.2	173	60.3	761	55.3
Married	404	24.3	69	24.0	335	24.4
Separated	39	2.3	3	1.1	36	2.6
Cohabitate with a female partner	6	0.4	0	0.0	6	0.4
Cohabitate with a male partner	198	11.9	31	10.8	167	12.1
Divorced	79	4.8	11	3.8	68	4.9
Widower	3	0.2	0	0.0	3	0.2
Occupation						
Freelance	711	42.8	132	46.8	579	42.5
Own business	101	6.1	17	6.0	84	6.2
Farmer	18	1.1	4	1.4	14	1.0
Worker	121	7.3	23	8.2	98	7.2
Cosmetology	35	2.1	9	3.2	26	1.9
College student	47	2.8	7	2.5	40	2.9
Unemployed	12	0.7	0	0.0	12	0.9
Office worker	399	24.0	54	19.2	345	25.3
Catering	130	7.8	21	7.5	109	8.0
Other	72	4.3	15	5.3	57	4.2
Most frequent venue for meeting sex partners						
Internet	806	48.5	147	52.5	659	49.0
Hotel	72	4.3	14	5.0	58	4.3
Pub	44	2.6	9	3.2	35	2.6
Club	9	0.5	0	0.0	9	0.7
Public bath	112	6.7	13	4.6	99	7.4
Public Toilet	13	0.8	5	1.8	8	0.6
Park	156	9.4	31	11.1	125	9.3
Gay club	27	1.6	6	2.1	21	1.6
Other	385	23.2	55	19.6	330	24.6

The proportion of total participants indicated for PrEP when applying different guidelines ranged from 33∙4% (USPHSC, n = 556) to 94∙3% (KSA, n = 1296) (Table 3). Across the guidelines, sensitivity (i.e., the proportion of eventually infected MSM identified as PrEP eligible) increased as specificity (i.e., the proportion of non‐seroconverting MSM identified as not PrEP eligible) decreased. The USPHSC guidelines had the lowest sensitivity (43∙6%, 95% CI: 37∙7 to 49.5) and highest specificity (68∙7%, 95%CI: 66∙2 to 71∙1) and the KSA guidelines had the highest sensitivity (97∙2%, 95%CI: 94∙6 to 98∙8) and lowest specificity (6∙3%, 95%CI: 5∙0 to 7∙7). Compared to random allocation, sensitivity ranged from slightly worse (−4∙7% relative change, USPHSR) to 30∙2% (relative percent change) better than random (USPHSC). However, in absolute terms, none of the sensitivity values increased by more than 11% when compared with random allocation. For example the EACS guidelines had the largest absolute difference in sensitivity, from 39∙4% using random allocation to 50∙2% using the guidelines. Across all guidelines, specificity was not meaningfully better than random allocation of PrEP.

**Table 3:** Confusion matrix values for the performance of international PrEP eligibility guidelines among a cohort of MSM enrolled from 2009 to 2016, You’An Hospital, Beijing, China

Guidelines	n	Sensitivity	Specificity	Positive predictive value	Negative predictive value
EACS (95% CI)	657	0.502 (0.442 to 0.561)	0.627 (0.601 to 0.653)	0.219 (0.188 to 0.253)	0.858 (0.835 to 0.879)
Random (95% BI)	657	0.394 (0.345 to 0.446)	0.605 (0.594 to 0.616)	0.172 (0.151 to 0.195)	0.827 (0.813 to 0.842)
KSA (95% CI)	1569	0.972 (0.946 to 0.988)	0.063 (0.050 to 0.077)	0.178 (0.159 to 0.198)	0.915 (0.839 to 0.963)
Random (95% BI)	1569	0.944 (0.920 to 0.969)	0.057 (0.052 to 0.062)	0.173 (0.168 to 0.177)	0.830 (0.755 to 0.904)
SA (95% CI)	1296	0.840 (0.792 to 0.880)	0.233 (0.211 to 0.257)	0.186 (0.165 to 0.208)	0.875 (0.836 to 0.907)
Random (95% BI)	1296	0.780 (0.735 to 0.822)	0.221 (0.211 to 0.230)	0.173 (0.163 to 0.182)	0.828 (0.793 to 0.861)
Taiwan (95% CI)	918	0.624 (0.565 to 0.680)	0.463 (0.436 to 0.490)	0.195 (0.170 to 0.222)	0.855 (0.828 to 0.880)
Random (95% BI)	918	0.551 (0.498 to 0.603)	0.448 (0.437 to 0.459)	0.172 (0.156 to 0.188)	0.827 (0.807 to 0.847)
UK (95% CI)	849	0.533 (0.474 to 0.592)	0.494 (0.467 to 0.521)	0.180 (0.155 to 0.208)	0.835 (0.808 to 0.860)
Random (95% BI)	849	0.512 (0.456 to 0.564)	0.490 (0.478 to 0.501)	0.173 (0.154 to 0.191)	0.828 (0.808 to 0.846)
USPHSC (95% CI)	556	0.436 (0.377 to 0.495)	0.687 (0.662 to 0.711)	0.225 (0.191 to 0.262)	0.854 (0.831 to 0.874)
Random (95% BI)	556	0.334 (0.286 to 0.383)	0.666 (0.656 to 0.676)	0.173 (0.147 to 0.198)	0.827 (0.815 to 0.840)
Random (95% BI)	1244	0.749 (0.700 to 0.794)	0.252 (0.242 to 0.262)	0.173 (0.162 to 0.183)	0.828 (0.795 to 0.859)
WHO (95% CI)	734	0.544 (0.484 to 0.602)	0.580 (0.553 to 0.606)	0.213 (0.184 to 0.244)	0.859 (0.835 to 0.881)
Random (95% BI)	734	0.439 (0.390 to 0.495)	0.558 (0.548 to 0.570)	0.172 (0.153 to 0.193)	0.827 (0.812 to 0.844)

BI, bootstrap interval; CI, confidence interval.

The EACS guidelines demonstrated the highest Matthew’s Correlation Coefficient (MCC = 0∙100) and Youden’s Index (YI = 0∙129) values. Commonly used interpretation guidelines consider any correlation coefficient below 0∙3 to be negligible or not meaningful correlation [28]. The KSA guidelines had the highest diagnostic odds ratio (DOR = 2∙323) (Table 4) and according to previous guidance, diagnostic tests that have likelihood ratio performance measures below 3 rarely alter clinical decisions [29]. The EACS, KSA, SA and WHO guidelines all had similar values for the F1 score (range: 0∙301 to 0∙306). The USPHSR score‐based criteria performed worse than random allocation of PrEP across all binary classification performance measures.

**Table 4:** Binary classification performance statistics for international PrEP eligibility guidelines among a cohort of MSM enrolled from 2009 to 2016, You’An Hospital, Beijing, China

Guidelines	n	F1 score	Matthews correlation coefficient			Youden's index			Diagnostic odds ratio		
EACS (95% CI)	657	0.305	0.100			0.129			1.694		
Random (95% BI)	657	0.239 (0.210 to 0.271)	−0.001	−0.047	0.048	−0.002	−0.061	0.062	0.993	0.772	1.289
KSA (95% CI)	1569	0.301	0.057			0.035			2.323		
Random (95% BI)	1569	0.292 (0.284 to 0.300)	0.002	−0.047	0.050	0.001	−0.029	0.030	1.018	0.624	2.034
SA (95% CI)	1296	0.305	0.067			0.073			1.594		
Random (95% BI)	1296	0.283 (0.267 to 0.298)	0.001	−0.049	0.047	0.001	−0.053	0.052	1.008	0.745	1.380
Taiwan (95% CI)	918	0.297	0.066			0.087			1.428		
Random (95% BI)	918	0.262 (0.237 to 0.287)	−0.001	−0.049	0.047	−0.002	−0.065	0.061	0.993	0.770	1.285
UK (95% CI)	849	0.269	0.021			0.027			1.116		
Random (95% BI)	849	0.259 (0.231 to 0.285)	0.002	−0.049	0.049	0.002	−0.065	0.065	1.008	0.770	1.300
USPHSC (95% CI)	556	0.297	0.098			0.122			1.692		
Random (95% BI)	556	0.228 (0.195 to 0.261)	0.000	−0.047	0.047	0.000	−0.059	0.059	1.001	0.761	1.296
USPHSR (95% CI)	1244	0.268	−0.036			−0.041			0.811		
Random (95% BI)	1244	0.281 (0.263 to 0.298)	0.001	−0.050	0.049	0.001	−0.058	0.056	1.007	0.746	1.369
WHO (95% CI)	734	0.306	0.094			0.124			1.644		
Random (95% BI)	734	0.247 (0.219 to 0.278)	−0.002	−0.047	0.049	−0.003	−0.062	0.065	0.989	0.776	1.297

BI, bootstrap interval; CI, confidence interval.

## 4 Discussion

These results indicate that none of the currently established international PrEP guidelines performed well in identifying HIV seroconversions among a hospital‐based cohort of MSM in Beijing. The ability to correctly identify participants that would seroconvert improved as the number of men who were indicated for PrEP increased, but at the cost of a loss of specificity. When compared to random allocation of PrEP, the sets of guidelines that demonstrated the largest relative improvement (e.g. EACS, WHO, USPHSC) only identified about half of new HIV cases. These results align with previous arguments that current existing behavioural PrEP indication guidance may be insufficient for use across different populations [30]. Additionally, the results from this analysis could contribute to informing future policy decisions related to HIV prevention in China. Although there are not currently any national guidelines on the use of TDF/FTC for HIV prevention in China, a PrEP implementation study has recently been launched [31]. That multisite study aims to collect comprehensive data on the PrEP use cascade in order to develop guidance on PrEP implementation. The development of PrEP eligibility guidelines will be an essential component of any implementation programme.

In this analysis, we provide an innovative method of assessing the performance of a set of clinical guidelines through a retrospective cohort analysis. Typically, analysis of the performance of clinical diagnostic guidelines rely on the use of area under the curve (AUC) test statistics, which can be difficult for clinicians and decision makers to interpret. Here, we present several pieces of the confusion matrix (i.e. contingency table that reports false positives, false negatives, true positives and true negatives), which allows readers to separate the impact of different guidelines on sensitivity and specificity. Additionally, the inclusion of binary classification performance metrics that are typically used in machine learning classification algorithms provide a more holistic understanding of each guideline performance. Finally, presenting the results as a comparison to random allocation of PrEP provides an intuitive baseline measure for decision makers to use when quantifying the additional benefit of potential guidelines. Future studies of this nature should incorporate a similar comparison point in order to directly compare, in an easily interpretable manner, the added benefit of diagnostic algorithms that differ in their scope.

Although the use of binary classification measures from external fields provides an innovative way to assess PrEP guidelines, each measure has individual limitations when used in this application. For example the F1 score places equal importance on sensitivity and positive predictive value, but in this context, false negatives (i.e. seroconversions that were not indicated for PrEP) resulting from low sensitivity are much more consequential. Similarly, Youden’s Index applies equal weight to false positives and false negatives even though the public health repercussions of failing to identify future seroconverts as PrEP eligible outweigh the provision of PrEP to some people that will not seroconvert. However, despite these individual limitations, the interpretation of several summary measures and statistics together improve our understanding of the performance of each set of guidelines.

Additionally, this study has important limitations. First, only MSM from You’an Hospital in Beijing, China were included in this analysis, which limits the generalizability of the performance of each set of guidelines. Similarly, data for this analysis were collected prior to March 2016 and it is possible the prevalence of risk behaviours or willingness to report risk behaviours may differ in 2020. As a comparison point, a recent cross‐sectional analysis published in 2019 used a modified version of the USPHC PrEP eligibility assessment tool and found 45.6% of surveyed MSM in China met the eligibility requirements [32]. Additional studies should replicate this approach in different settings to assess the appropriateness of a set of guidelines in their specific context. Second, although we had robust data on sexual behaviours and partners, these data were collected as part of a cohort study and not all data points were complete or analogous to each specific set of PrEP initiation guidelines. For example condom use frequency was missing for 197 participants (11∙9%) and we assumed those participants used condoms less than 100% of the time. This is a conservative approach, but the assumption could have potentially led to indicating some MSM for PrEP that would not have met the definition if they reported full condom use data. Similarly, we had data on partner HIV status, but we did not have data on partner treatment status or viral suppression. To operationalize the guidelines from South Africa, the UK and WHO, we assumed any reported HIV sex partners did not have confirmation of viral suppression. Data from China HIV/AIDS national information systems report that 42.7% of MSM with an HIV diagnosis was virally suppressed in 2015 [33], which means our approach may slightly overestimate the number of men indicated for PrEP using these three guidelines. Additionally, we did not have data on drug or alcohol use during sex or post‐exposure prophylaxis (PEP) and could not include the guideline criteria that referenced those behaviours. For guidelines that have criteria based on drug or alcohol use during sex (USPHSR, SA, Taiwan), this lack of data could have led to the misclassification of some MSM who should be indicated for PrEP. However, the impact of this misclassification on these results is expected to be minor because it would only arise among men who self‐reported the use of drugs during sex but did not report any of the other behaviours outlined in each set of guidelines. Furthermore, considering only 2.4% of Chinese MSM reported using crytal meth in the past 12 months in a recent study, we do not anticipate this misclassification to have a large impact in this context. Finally, it is not common practice in China to test MSM for gonorrhoea or chlamydia, so we only had self‐report data for those guideline criteria. It is possible the inclusion of additional lab data on sexually transmitted infections would change results, which would indicate that the accuracy and completeness of assessment data are a primary piece of the correct identification of men at high risk of seroconversion. This limitation, however, is also a strength: any large‐scale implementation of guidelines will be restricted to data collected in standard practice, such as those used in our data collection. Our validation dataset may therefore better represent the likely impact of guidelines adoption. Additionally, this highlights the importance of both developing a locally relevant eligibility algorithm and strengthening the healthcare system in which it is applied.

## 5 Conclusions

In conclusion, utilizing current international PrEP indication guidelines had little utility in correctly identifying Beijing MSM that would seroconvert to HIV. In high incidence settings, it may be best to indicate for PrEP all sexually active persons interested in adopting the prevention mechanism. Another guidance document from the World Health Organization endorses this idea, saying PrEP should be offered as a prevention choice to anyone at “substantial risk”, which is defined as an incidence greater than 3 per 100 person‐years [34]. Considering this guidance differs from the WHO implementation tool included in this analysis, it is not clear which should be the correct interpretation. To ensure PrEP prevention resources are maximized, improved clinical decision algorithms that incorporate additional data may produce better results, and should be pursued. However, in the interim, it is essential that individuals at high risk of transmission not be excluded from PrEP based on guidelines that perform only slightly better than chance.

## Competing Interests

No conflicts of interest to declare.

## Authors’ Contributions

EH conducted the analysis and wrote the first draft of the manuscript. LW helped compile the data, assisted with the analysis and interpret results. XH managed the data collection and helped interpret the results. PS helped interpret the results and critically revise the manuscript. AS secured funding for the study, interpreted results and critically revised the manuscript. All authors approved the final draft.
